# Neuroprotective effects of curcumin, memantine, and caffeic acid in a Rat model of cerebral ischemia-reperfusion injury

**DOI:** 10.3389/fphar.2026.1739566

**Published:** 2026-03-13

**Authors:** Celal Ozbek Cakir, Nihat Yumuşak, Muhammet Yasin Tekeli, Davut Bayram, Latife Cakir Bayram, Murat Baloğlu

**Affiliations:** 1 Department of Neurosurgery, Afyon Park Hayat Hospital, Afyon, Türkiye; 2 Department of Pathology, Faculty of Veterinary Medicine, Harran University, Şanlıurfa, Türkiye; 3 Department of Pharmacology and Toxicology, Faculty of Veterinary Medicine, Erciyes University, Kayseri, Türkiye; 4 Department of Animal Science, Division of Animal Science, Faculty of Veterinary Medicine, Erciyes University, Kayseri, Türkiye; 5 Department of Pathology, Faculty of Veterinary Medicine, Erciyes University, Kayseri, Türkiye; 6 Department of Neurosurgery, Eskişehir City Hospital, Eskişehir, Türkiye

**Keywords:** antioxidant defense, caffeic acid, cerebral ischemia-reperfusion injury, curcumin, memantine, neuronal apoptosis, neuroprotection, oxidative stress

## Abstract

Cerebral ischemia–reperfusion (I/R) injury leads to neuronal loss through oxidative stress, inflammation, and apoptosis. Curcumin, memantine, and caffeic acid possess antioxidant and neuroprotective properties. This study compared their efficacy in a rat model of transient cerebral ischemia. Forty male Wistar albino rats were initially allocated into six experimental groups; however, the vehicle control group was excluded from statistical analyses, and data are presented for five groups (n = 8 per group): sham, ischemia–reperfusion (I/R), I/R + curcumin (300 mg/kg/day), I/R + memantine (10 mg/kg/day), and I/R + caffeic acid (10 μmol/kg/day). Transient ischemia was induced by bilateral carotid artery occlusion for 30 min followed by 72 h reperfusion. Treatments were administered intraperitoneally beginning 30 min after reperfusion and continued once daily for five consecutive days. Histopathological and immunohistochemical analyses of the frontoparietal cortex demonstrated that I/R induced severe neuronal degeneration, necrosis, gliosis, vascular hyperemia, and marked inflammatory and apoptotic activation, with severity scores reaching the highest grade (3) (p < 0.001 vs. sham). Memantine and caffeic acid significantly reduced all degenerative, inflammatory, and apoptotic parameters (p < 0.001), restoring histopathological morphology to levels not statistically different from the sham group (p > 0.05). In particular, caspase-3, IL-1β, TNF-α, and TUNEL positivity were markedly suppressed in both treatment groups. In contrast, curcumin treatment resulted in only partial attenuation of neuronal degeneration and inflammatory infiltration, without achieving statistical significance in most parameters (p > 0.05 vs. I/R). Correlation analyses revealed strong negative associations between antioxidant enzyme activities (GR and GST) and histopathological damage scores (r = −0.71 to −0.82, p < 0.001), as well as strong positive correlations between apoptotic/inflammatory markers and neuronal injury severity (r = 0.68 to 0.79, p < 0.001). These findings demonstrate that memantine and caffeic acid exert robust histopathological neuroprotection against cerebral ischemia–reperfusion injury, whereas curcumin shows limited efficacy under the applied experimental conditions.

## 1 Introduction

Cerebral ischemia–reperfusion (I/R) injury remains one of the leading causes of neuronal death and neurological dysfunction in stroke and related neurovascular disorders. The pathophysiology of I/R injury is characterized by a complex interplay of glutamate-mediated excitotoxicity, intracellular calcium overload, mitochondrial dysfunction, oxidative stress, neuroinflammation, and activation of apoptotic signaling pathways (Lipton, 2006; Zhang et al., 2023; Rehman et al., 2024). Although reperfusion is essential for restoring cerebral blood flow, it paradoxically exacerbates tissue damage by amplifying oxidative stress and inflammatory responses, thereby intensifying excitotoxic and apoptotic processes. These interconnected mechanisms establish a self-sustaining cycle of injury that contributes to progressive neuronal degeneration and cognitive decline (Zhao et al., 2008; Jurcau and Ardelean, 2022; Rehman et al., 2024; Lv et al., 2025). Consequently, I/R injury disrupts cellular energy homeostasis and activates multiple cell death pathways, ultimately leading to irreversible brain damage (Majumder, 2024; Kumar Saini and Singh, 2024). In recent years, considerable research interest has been directed toward identifying neuroprotective compounds capable of targeting the multifactorial mechanisms underlying ischemic brain injury. In this context, memantine, an N-methyl-D-aspartate (NMDA) receptor antagonist (Bufan et al., 2024); curcumin, a bioactive polyphenol derived from *Curcuma longa*; and caffeic acid, a phenolic antioxidant, have emerged as compounds exhibiting neuroprotective potential due to their antioxidant, anti-inflammatory, and anti-apoptotic properties (Subedi and Gaire, 2021; Jurcau and Ardelean, 2022; Pichardo-Rojas et al., 2023).

Memantine, a clinically approved uncompetitive NMDA receptor antagonist, exerts its neuroprotective effects primarily by attenuating glutamate-mediated excitotoxicity through limiting pathological calcium influx into neurons. By reducing intracellular calcium overload, memantine preserves synaptic integrity and mitigates excitotoxic neuronal injury in experimental models of cerebral ischemia (Beladi Moghadam et al., 2021; Pichardo-Rojas et al., 2023; Sulimai et al., 2025).

Beyond its anti-excitotoxic action, recent evidence indicates that memantine enhances endogenous antioxidant defense systems and reduces ischemia-associated apoptotic signaling, thereby promoting neuronal survival following ischemia–reperfusion injury (Kocabeyoğlu et al., 2025). Moreover, memantine has been shown to attenuate neuroinflammatory responses and is associated with reduced TNF-α and IL-1β expression, supporting its role in limiting secondary inflammatory damage and attenuating neurovascular dysfunction after cerebral ischemia (Pichardo-Rojas et al., 2023; Danysz et al., 2025).

Curcumin is a naturally occurring polyphenol with well-established antioxidant and anti-inflammatory properties. It alleviates oxidative stress by reducing reactive oxygen species production and lipid peroxidation while supporting endogenous antioxidant defense systems (Yang et al., 2023; Moldoveanu et al., 2024; Hao et al., 2025). In experimental models of cerebral ischemia, curcumin has been reported to exert neuroprotective effects associated with reductions in inflammatory cytokines and apoptotic markers, including decreased caspase-3 activation and fewer TUNEL-positive cells (Zhang et al., 2023; Moldoveanu et al., 2024).

Caffeic acid is a naturally occurring phenolic compound with well-established antioxidant and anti-inflammatory properties that contribute to its neuroprotective potential in cerebral ischemia–reperfusion injury. By neutralizing reactive oxygen species, caffeic acid reduces lipid peroxidation and limits oxidative damage in ischemic brain tissue (Li et al., 2024). Experimental studies have further demonstrated that caffeic acid is associated with decreased inflammatory cytokine expression and reduced apoptotic activity, suggesting protective effects against ischemia–reperfusion–induced neuronal injury (Pérez et al., 2023; Li et al., 2024).

Experimental studies in models of cerebral ischemia have reported that caffeic acid is associated with decreased inflammatory cytokine expression, reduced caspase-3 activation, and fewer TUNEL-positive cells. These findings suggest that caffeic acid may limit secondary inflammatory responses and apoptotic cell death following ischemia–reperfusion injury (Pérez et al., 2023; Li et al., 2025).

In experimental models of cerebral ischemia–reperfusion injury, evaluation of oxidative stress and antioxidant parameters, together with histopathological and immunohistochemical evaluation of inflammatory and apoptotic responses, is commonly used to assess neuroprotective effects. Correlation analyses between oxidative stress indices and tissue injury severity are also applied to examine the relationship between biochemical alterations and histopathological damage in ischemic brain injury (Rehman et al., 2024; Yoo et al., 2025; Zhang et al., 2023).

These compounds have been shown to attenuate ischemic brain injury by reducing oxidative stress, suppressing pro-inflammatory cytokines, and limiting apoptotic mechanisms (Kwon et al., 2021; Cabalar et al., 2022; Zhang et al., 2023). Given the multifactorial nature of cerebral ischemia, which involves oxidative stress, inflammation, and apoptosis, therapeutic strategies increasingly focus on compounds capable of targeting these processes simultaneously. In this context, curcumin, memantine, and caffeic acid are regarded as noteworthy compounds due to their distinct yet potentially complementary neuroprotective mechanisms.

However, despite extensive individual investigation of these compounds, direct comparative evaluations under standardized ischemia–reperfusion conditions integrating histopathological and molecular parameters remain limited. The present study therefore aimed to systematically and comparatively evaluate the neuroprotective effects of these compounds through a comprehensive assessment encompassing histopathological, immunohistochemical, and oxidative stress analyses performed within the same anatomical region.

## 2 Materials and methods

### 2.1 Animals and experimental design

The experimental protocol was approved by the Erciyes University Local Ethics Committee on Animal Experiments (protocol code 20/023, dated 8 January 2020). All procedures were conducted in accordance with national legislation governing the protection of experimental animals and were consistent with Directive 2010/63/EU of the European Parliament on the protection of animals used for scientific purposes. The study was reported in compliance with the ARRIVE guidelines Forty male Wistar albino rats (6–8 weeks old, 190–220 g) were housed under standard laboratory conditions (12 h light/12 h dark cycle, 22 °C ± 2 °C temperature, 45%–55% relative humidity). Rats had *ad libitum* access to standard pellet chow and water throughout the study. The animals were randomly assigned to five groups (n = 8 per group): a sham group, an ischemia-reperfusion (I/R) group, an I/R + curcumin group, an I/R + memantine group, and an I/R + caffeic acid group.

Animals were initially randomly allocated to six groups, but the I/R + vehicle group was excluded from inferential analysis because early checks showed no deviation from baseline. All n values referred to in the reports refer to animals incorporated in final analyses.

Histopathological evaluations were performed on coronal brain sections corresponding to bregma −1.8 to −3.0 mm, including the frontoparietal cortex, a region known to be highly susceptible to ischemic injury (Subedi and Gaire, 2021; Cabalar et al., 2022). To reduce regional heterogeneity and maintain anatomically comparable analysis across all animals, tissue sampling was restricted to the defined cortical area (layers II–VI). The selected cortical region demonstrated consistent patterns of neuronal degeneration and inflammatory infiltration. The sampling level is shown schematically in Supplementary Figure S1.

### 2.2 Experimental groups

The experimental design comprised six groups, as summarized in Table 1.

**TABLE 1:** Experimental groups and treatment protocols.

Group No	Group name	Sample size	Description
1	Sham	8	Surgical incision only; no ischemia or treatment
2	Ischemia-reperfusion (I/R) control	8	Ischemia-reperfusion was performed, but no treatment or vehicle was administered
3	Ischemia-reperfusion + vehicle (DMSO) group	8	Vehicle was administered following ischemia-reperfusion
4	I/R + curcumin	8	Curcumin was administered at a dose of 300 mg/kg following ischemia-reperfusion
5	I/R + memantine	8	Memantine was administered at a dose of 10 mg/kg following ischemia-reperfusion
6	I/R + caffeic acid	8	Caffeic acid was administered at a dose of 10 μmol/kg following ischemia-reperfusion

A total of forty-eight male Wistar albino rats (6–8 weeks old, 190–220 g) were included in the study and randomly assigned to six groups (n = 8 per group): sham, I/R, I/R + vehicle, I/R + curcumin, I/R + memantine, and I/R + caffeic acid.

Animals in the sham group underwent anesthesia and surgical exposure without middle cerebral artery occlusion.

No animals died following ischemia induction or during the 72-h reperfusion period. All animals were euthanized 72 h after reperfusion and included in the histopathological, immunohistochemical, TUNEL, and biochemical evaluations.

Although a vehicle control group was included to ensure methodological consistency, preliminary analyses revealed no significant differences compared to the I/R group; therefore, these data were not included in the final statistical comparisons. A total of forty-eight male Wistar albino rats (6–8 weeks old, 190–220 g) were included in the study and randomly assigned to six groups (n = 8 per group): sham, I/R, I/R + vehicle, I/R + curcumin, I/R + memantine, and I/R + caffeic acid. No animals died following ischemia induction or during the 72-h reperfusion period. All animals included in the study were euthanized 72 h after reperfusion. All animals were included in the histopathological, immunohistochemical, TUNEL, and biochemical evaluations.

Although a vehicle control group was included to ensure methodological integrity, preliminary analyses revealed no significant differences from baseline; therefore, data from this group were not included in the statistical analyses.

### 2.3 Drugs and pharmacological administration

In this study, caffeic acid (≥98.0%, Sigma, C0625-5G), curcumin (derived from Curcuma longa, Sigma, C1386-5G), and memantine (Melanda®, Ali Raif, Turkey) were used. All compounds were dissolved directly in 0.1% dimethyl sulfoxide (DMSO; Sigma, St. Louis, MO, United States) without further dilution (Santos et al., 2003). Drug administrations were initiated 30 min after reperfusion and continued once daily for five consecutive days via intraperitoneal (IP) injection.

The administered doses were selected based on previously published experimental studies. Curcumin was administered at 300 mg/kg/day according to Hocking et al. (2018). Memantine was administered at 10 mg/kg/day based on Creeley et al. (2006). Caffeic acid phenethyl ester was administered at 10 μmol/kg/day as described by Rifaioglu et al. (2015). DMSO was used as the sole solvent throughout the treatment period to ensure complete solubility and systemic bioavailability of the compounds.

### 2.4 Induction of cerebral ischemia-reperfusion injury

Rats were anesthetized with ketamine hydrochloride (60 mg/kg) (Ketasol® 10%, Richter Pharma AG, Wels, Austria) and xylazine (6 mg/kg) administered intramuscularly. Depth of anesthesia was assessed every 10 min by monitoring the plantar withdrawal response and palpebral reflex. Supplemental ketamine (30 mg/kg) was administered when required. Core body temperature was monitored using a rectal probe and maintained at 37.5 °C–38.5 °C.

Animals were placed in the supine position under aseptic conditions, and a midline cervical incision was performed. Using blunt and sharp microdissection techniques, both common carotid arteries were carefully isolated while preserving surrounding tissues. Global cerebral ischemia was induced by applying Yasargil aneurysm clips to both common carotid arteries for 30 min. Following clip removal, reperfusion was allowed for 72 h (Otawara et al., 2009).

At the end of the surgical procedure, the incision was sutured, and animals were returned to their cages for postoperative monitoring. Experimental treatments were administered according to the assigned experimental groups.

### 2.5 Neurological assessment

Behavioral assessment was conducted using neurological deficit scoring, employing a 5-point neurological deficit scale widely used in rodent models of cerebral ischemia–reperfusion (Montoya García and Quintanar, 2020; Ruan and Yao, 2020). Neurological deficits were evaluated at 24 and 72 h following reperfusion based on motor responses, postural alterations, coordination, gait abnormalities, and level of consciousness. Scores were defined as follows: 0 = no deficit; 1 = mild forelimb weakness or postural asymmetry; 2 = severe forelimb weakness or circling; 3 = falling or marked loss of coordination; 4 = loss of walking ability or decreased level of consciousness; and 5 = death. All evaluations were performed in a blinded manner by an investigator unaware of the experimental group assignments.

### 2.6 Biochemical analysis of oxidative stress markers

Brain tissues were collected and stored at −80 °C until analysis. Oxidative stress markers and antioxidant parameters were assessed using standard biochemical methods. Total protein concentration was determined by the Lowry method (Lowry et al., 1951). Malondialdehyde (MDA), an index of lipid peroxidation, was measured according to the method of Ohkawa et al. (1978). Reduced glutathione (GSH) levels were determined using the Sedlak and Lindsay method (Sedlak and Lindsay, 1968). Enzymatic antioxidant activities were measured as follows: glutathione peroxidase (GSH-Px) by the method of Paglia and Valentine (1967), glutathione reductase (GR) by Carlberg and Mannervik (1985), glutathione S-transferase (GST) by Habig et al. (1974), superoxide dismutase (SOD) by Sun et al. (1988), and catalase (CAT) by the method of Lück (Lück, 1965).

### 2.7 Histopathological examination

Rats were euthanized by an overdose of sodium pentobarbital administered intraperitoneally in accordance with AVMA Guidelines (2020). The whole brain was immediately removed and fixed in 10% neutral-buffered formalin, processed through graded alcohol and xylene, and embedded in paraffin. Sections of 5 µm thickness were stained with Hematoxylin and Eosin (HxE) and evaluated for neuronal degeneration, necrosis, gliosis, and vascular changes.

Histopathological findings were evaluated semi-quantitatively using an ordinal grading system ranging from 0 (no lesion) to 3 (severe lesion), as previously described (Manning et al., 2008; Gibson-Corley et al., 2013). In this system, scores reflect increasing lesion extent and severity based on morphological criteria. Each parameter was scored independently and expressed as median (IQR) values. Given the ordinal nature of the data, non-parametric statistical analyses were applied.

### 2.8 Immunohistochemical staining

Paraffin-embedded brain sections (4 µm thick) were immunostained using the Avidin–Biotin Complex (ABC) method.Antigen retrieval was performed in citrate buffer (pH 6.0), followed by blocking and incubation with the following primary antibodies: Caspase-3 (Invitrogen, PA5-16335), TNF-α (Abcam, ab6671), and IL-1β (Invitrogen, PA5-88078). Antibody dilutions are provided in Table 2. Detection was carried out using 3,3′-diaminobenzidine (DAB) as the chromogen, and sections were counterstained with hematoxylin. Immunoreactivity was semi-quantitatively scored as follows: 0 (<1%), 1 (1%–25%), 2 (26%–75%), and 3 (>75%).

**TABLE 2:** Antibodies used in the study, their brands and dilution rates.

Monoclonal/Polyclonal	Company (make)	Catalog number	Target antigen	Target species	Dilution and incubation
Polyclonal	Invitrogen, Thermo Fisher scientific Rockford, United States	PA5-16335	Caspase 3	Dog, Rabbit, Rat, human, Mouse	1/100–1 h
Polyclonal	Abcam Cambridge United Kingdom	ab6671	Anti-TNF alpha	Human Mouse Rat	1/100–1 h
Polyclonal	Invitrogen, Thermo Fisher scientific Rockford, United States	PA5-88078	IL-1β	Human Mouse Rat	1/200–1 h

### 2.9 TUNEL assay

Apoptotic cells were detected using the TUNEL (Terminal deoxynucleotidyl transferase dUTP nick end labeling) method, following the manufacturer’s instructions for the *In Situ* Cell Death Detection Kit (POD, Roche, Germany). After deparaffinization, tissue sections were treated with Proteinase K and incubated with the TUNEL reaction mixture. After incubation with a POD-conjugated anti-fluorescein antibody, labeling was demonstrated using 3,3′-diaminobenzidine (DAB) as the chromogen. Sections were counterstained with hematoxylin.

### 2.10 Statistical analysis

All statistical analyses were performed using IBM SPSS Statistics version XX (IBM Corp., Armonk, NY, United States). Data were expressed as median [interquartile range (IQR)] due to the non-parametric distribution verified by the Shapiro–Wilk normality test.

Group differences for histopathological scores (degeneration, necrosis, neuronophagia, gliosis, astrogliosis/microgliosis, and hyperemia) and immunohistochemical/TUNEL scores (IL-1β, TNF-α, Caspase-3, and TUNEL-positive nuclei) were analyzed using the Kruskal–Wallis test. When a significant group effect was detected (p < 0.05), Dwass–Steel–Critchlow–Fligner (DSCF) *post hoc* multiple comparisons were applied to identify pairwise differences among groups. No Bonferroni or Holm corrections were applied, as the DSCF test provides an intrinsic non-parametric adjustment for multiple testing. The significance level was set at p < 0.05 for all analyses.

## 3 Results

### 3.1 Histopathological evaluation

**FIGURE 1:**
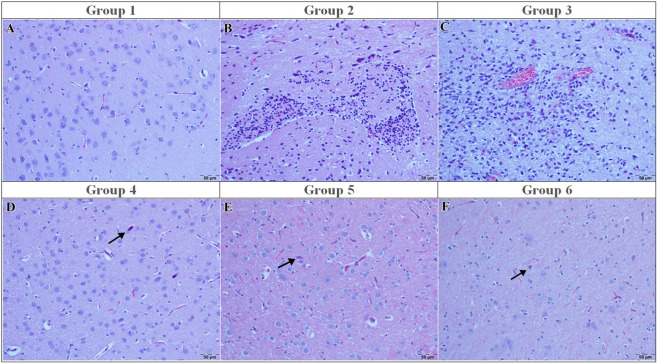
Light micrographs of hematoxylin and eosin–stained sections from the experimental groups. **(A)** Sham: Intact cortical lamination with normal neuronal morphology; **(B)** I/R: Neuronal degeneration and necrosis accompanied by gliosis and inflammatory cell infiltration; **(C)** I/R + vehicle: Neuronal degeneration and vascular hyperemia comparable to the I/R group; **(D)** I/R + curcumin: Neuronal degeneration (arrow); **(E)** I/R + memantine: Neuronal degeneration with inflammatory cell infiltration (arrow); **(F)** I/R + caffeic acid: Degenerative neurons (arrow) with preserved cortical organization. H&E. Scale bar = 50 μm.

In the sham group (Group 1), the brain histopathology appeared normal, with no evidence of neuronal degeneration, necrosis, gliosis, vascular hyperemia, or inflammatory infiltration (all median scores: 0 [0–0]) (Figure 1A).

In contrast, the ischemia-reperfusion (I/R) control group (Group 2) exhibited severe cortical injury characterized by neuronal degeneration, neuronophagia, neuronal necrosis, prominent gliosis and astrogliosis, intense vascular hyperemia, and marked inflammatory cell infiltration (all median scores: 3 [3–3], p < 0.001 vs. sham), representing the highest injury category within this semi-quantitative grading system (Figure 1B). Microscopically, numerous degenerative neurons and marked gliosis were observed. Similarly, in the I/R+ vehicle (DMSO) group (Group 3), brain tissues were showed vascular hyperemia, neuronal necrosis and gliosis (Figure 1C)

Similarly, the I/R + curcumin group (Group 4) demonstrated comparable injury severity (median: 3 [3–3], p > 0.05 vs. I/R). Neuronal degeneration remained at 3 [3–3], and inflammatory infiltration as well as gliosis were not significantly reduced. Histologically, numerous degenerative neurons and persistent gliosis were observed (Figure 1D).

In contrast, memantine treatment (Group 5) significantly reduced the severity of pathological findings compared to the I/R group. Neuronal degeneration scores decreased from 3 [3–3] to 0 [0–0] (p < 0.001). Necrosis, neuronophagia, gliosis, and vascular hyperemia followed the same pattern (all median: 0 [0–0], p < 0.001 vs. I/R). Microscopically, only a few degenerative neurons and minimal vascular hyperemia were observed, with preservation of normal cortical layering (Figure 1E).

Similarly, in the caffeic acid group (Group 6), neuronal degeneration decreased from 3 [3–3] to 0 [0–0] (p < 0.001), and inflammatory infiltration and gliosis were also reduced to 0 [0–0], reaching levels comparable to the sham group (p > 0.05 vs. sham). Histologically, very few degenerative neurons and minimal inflammatory changes were observed (Figure 1F).

Overall, neuronal degeneration, necrosis, neuronophagia, focal gliosis, astrogliosis/microgliosis, and vascular hyperemia were significantly elevated in Groups 2 and 3 compared to Groups 1, 4, and 5 (all p < 0.001). Groups 5 and 6 did not differ significantly from the sham group (p > 0.05). Complete histopathological scoring results are presented in Table 3.

**TABLE 3:** Histopathological, immunohistochemical, and TUNEL scoring results.

Parameter	Sham (G1)	I/R (G2)	I/R + CurCumin (G3)	I/R + Memantine (G4)	I/R + caffeic acid (G5)	Kruskal–Wallis H (4)	P value	Significant DSCF comparisons
Degenerated neurons	0 (0–0)	3 (3–3)	3 (3–3)	0 (0–0)	0 (0–1)	35.8	<0.001	G2,G3 > G1,G4,G5
Necrosed neurons	0 (0–0)	3 (2–3)	3 (3–3)	0 (0–0)	0 (0–1)	34.9	<0.001	G2,G3 > G1,G4,G5
Neuronophagia	0 (0–0)	2 (2–2)	2 (2–2)	0 (0–0)	0 (0–0)	32.6	<0.001	G2,G3 > G1,G4,G5
Focal gliosis	0 (0–0)	3 (3–3)	3 (2–3)	0 (0–1)	0 (0–0)	33.2	<0.001	G2,G3 > G1,G4,G5
Astrogliosis/Microgliosis	0 (0–0)	2 (2–3)	3 (3–3)	0 (0–0)	0 (0–0)	31.5	<0.001	G2,G3 > G1,G4,G5
Hyperemia	0 (0–0)	3 (3–3)	3 (3–3)	0 (0–0)	0 (0–0)	34.4	<0.001	G2,G3 > G1,G4,G5
IL-1β	0 (0–1)	3 (3–3)	3 (3–3)	0 (0–0)	0 (0–0)	33.7	<0.001	G2,G3 > G1,G4,G5
TNF-α	0 (0–0)	3 (2–3)	3 (2–3)	0 (0–0)	0 (0–0)	32.9	<0.001	G2,G3 > G1,G4,G5
Caspase-3	0 (0–0)	3 (2–3)	3 (3–3)	0 (0–0)	0 (0–1)	32.1	<0.001	G2,G3 > G1,G4,G5
TUNEL	0 (0–0)	3 (3–3)	3 (3–3)	0 (0–0)	0 (0–1)	31.9	<0.001	G2,G3 > G1,G4,G5

Values are expressed as median [IQR]; n = 8 per group. Data were analyzed using the Kruskal–Wallis test followed by Dwass–Steel–Critchlow–Fligner *post hoc* comparisons. Statistical significance was defined as p < 0.05.

### 3.2 Immunohistochemical evaluation of pro-inflammatory cytokines IL-1β and TNF-α

**FIGURE 2:**
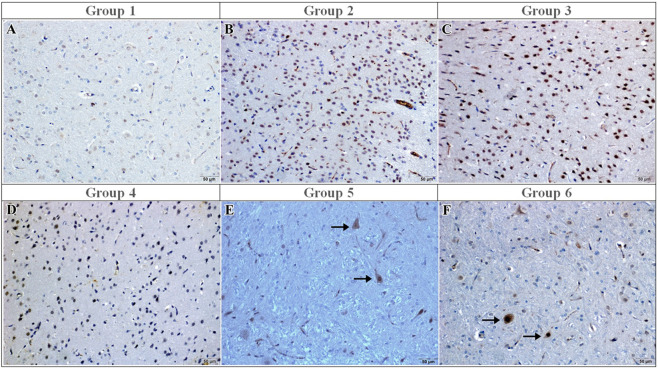
IL-1β immunoreactivity in the experimental groups. **(A)** Sham: Few IL-1β–immunopositive cells; **(B)** I/R: Strong IL-1β immunoreactivity with numerous positively stained cells; **(C)** I/R + vehicle: IL-1β immunoreactivity similar in extent and intensity to the I/R group; **(D)** I/R + curcumin: Moderate IL-1β immunoreactivity observed in a subset of cortical cells; **(E)** I/R + memantine: Mild IL-1β immunoreactivity with a reduced number of immunopositive cells (arrows); **(F)** I/R + caffeic acid: Weak IL-1β immunoreactivity, confined to scattered immunopositive cells (arrows). Immunoperoxidase method using 3,3′-diaminobenzidine (DAB) as chromogen and counterstained with hematoxylin. Scale bar = 50 μm.

IL-1β immunoreactivity was absent in the sham group (median: 0 [0–0]) (Figure 2A). In contrast, both the I/R and vehicle groups exhibited strong IL-1β immunoreactivity (median: 3 [3–3], p < 0.001 vs. sham), with numerous immunopositive neurons observed microscopically (Figure 2C). Curcumin treatment did not significantly reduce IL-1β immunoreactivity (median: 3 [3–3], p > 0.05 vs. I/R) (Figure 2D), and IL-1β immunoreactivity was not significantly different from the I/R group (Figure 2B). In contrast, memantine and caffeic acid treatments reduced IL-1β scores to 0 [0–0] (p < 0.001 vs. I/R), with only a few IL-1β–positive neurons observed (Figures 2E,F).

**FIGURE 3:**
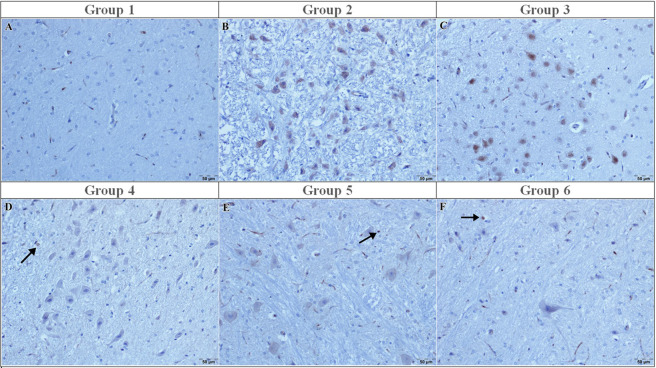
TNF-α immunoreactivity in the experimental groups. **(A)** Sham: Few TNF-α–immunopositive cells; **(B)** I/R: Strong TNF-α immunoreactivity with numerous positive cells; **(C)** I/R + vehicle: Increased TNF-α immunoreactivity comparable to the I/R group; **(D)** I/R + curcumin: Moderate TNF-α immunoreactivity (arrow); **(E)** I/R + memantine: Mild TNF-α immunoreactivity (arrow); **(F)** I/R + caffeic acid: Minimal TNF-α immunoreactivity (arrow). Immunoperoxidase method using 3,3′-diaminobenzidine (DAB) as chromogen and counterstained with hematoxylin. Scale bar = 50 μm.

TNF-α immunoreactivity was negative in the sham group (0 [0–0]) (Figure 3A) and markedly increased in the I/R and vehicle groups (3 [3–3], p < 0.001 vs. sham), with numerous TNF-α–positive neurons detected (Figure 3C). Curcumin treatment did not significantly reduce TNF-α immunoreactivity (median: 3 [3–3], p > 0.05 vs. I/R) (Figure 3D), and TNF-α immunoreactivity was not significantly different from the I/R group (Figure 3B). In contrast, memantine and caffeic acid reduced TNF-α scores to 0 [0–0] (p < 0.001 vs. I/R), with minimal TNF-α immunostaining observed (Figures 3E,F).

### 3.3 Immunohistochemical evaluation of apoptosis markers Caspase-3 and TUNEL

**FIGURE 4:**
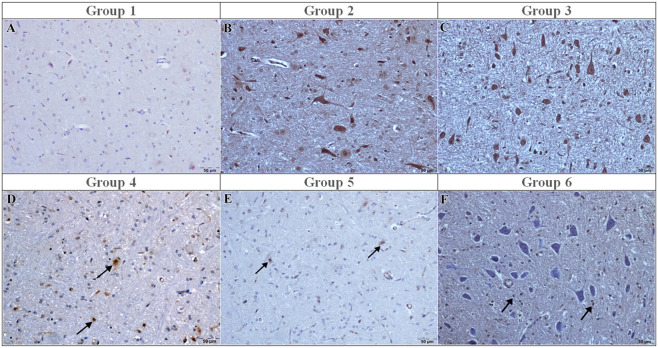
Caspase-3 immunoreactivity in the experimental groups. **(A)** Sham: Few Caspase-3–immunopositive cells; **(B)** I/R: Strong caspase-3 immunoreactivity in a large number of neurons; **(C)** I/R + vehicle: Increased Caspase-3 immunoreactivity comparable to the I/R group; **(D)** I/R + curcumin: Moderate Caspase-3 immunoreactivity (arrows); **(E)** I/R + memantine: Mild Caspase-3 immunoreactivity (arrows); **(F)** I/R + caffeic acid: Minimal Caspase-3 immunoreactivity (arrows). Immunoperoxidase method using 3,3′-diaminobenzidine (DAB) as chromogen and counterstained with hematoxylin. Scale bar = 50 μm.

Caspase-3 immunoreactivity was not detected in the sham group (0 [0–0]) (Figure 4A). Strong caspase-3 immunoreactivity was detected in the I/R and vehicle groups (3 [3–3], p < 0.001 vs. sham) (Figure 4C), with numerous immunopositive neurons observed. Curcumin treatment did not significantly reduce caspase-3 immunoreactivity (median: 3 [3–3], p > 0.05 vs. I/R) (Figure 4D), and Caspase-3 immunoreactivity was not significantly different from the I/R group (Figure 4B) . In contrast, memantine and caffeic acid reduced caspase-3 scores to 0 [0–0] (p < 0.001 vs. I/R), with only a few positive neurons detected (Figures 4E,F).

**FIGURE 5:**
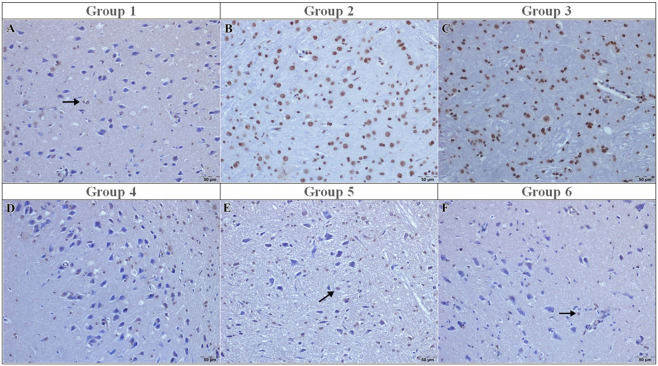
**(A)** TUNEL-negative nuclei in the sham group; **(B)** Strong nuclear TUNEL reactivity in the I/R group; **(C)** Many TUNEL-positive cells in the I/R + vehicle group; **(D)** Mild nuclear TUNEL staining in the I/R + curcumin group; **(E)** Few TUNEL-positive nuclei (arrow) in the I/R + memantine group; **(F)** Minimal TUNEL-positive cells in the I/R + caffeic acid group. TUNEL staining for detection of apoptotic nuclei. Scale bar = 50 μm.

TUNEL staining revealed no apoptotic nuclei in the sham group (0 [0–0]) (Figure 5A). The I/R and vehicle groups exhibited maximal apoptotic scores (3 [3–3], p < 0.001 vs. sham) (Figure 5C), characterized by numerous TUNEL-positive nuclei. Curcumin treatment was not significantly different from the I/R group (median: 3 [3–3], p > 0.05 vs. I/R) (Figures 5B,D). In contrast, memantine and caffeic acid significantly reduced TUNEL scores to 0 [0–0] (p < 0.001 vs. I/R) (Figures 5E,F).

Complete immunohistochemical and TUNEL scoring results (median and IQR values) are presented in Table 3.

### 3.4 Oxidative stress markers and antioxidant enzyme activities

No statistically significant between-group differences were observed for MDA, SOD, CAT, GSH-Px, GSH, or GST activities (all p > 0.05) (Table 4).

**TABLE 4:** Oxidative stress and antioxidant enzyme levels (median [IQR]).

Parameter	Sham	I/R	I/R + Curcumin	I/R + Memantine	I/R + Caffeic acid	Significant change	Direction
MDA (nmol/mg-protein)	2.032	2.083	2.197	1.904	2.591	—	—
SOD (U/mg-protein)	1.021	0.987	0.980	1.169	1.289	—	—
CAT (k/g-protein)	7.892	7.413	4.512	6.037	5.779	—	—
GSH-PX (nmol/min/g)	18.057	15.436	16.315	17.554	20.839	—	—
GSH (nmol/mg-protein)	133.0	125.9	127.2	141.0	151.4	—	—
GR (nmol/min/g)	153.4	21.7	22.9	21.8	30.2	Yes (all)	↓ sharp
GST (nmol/min/g)	86.8	80.9	73.5	123.7	123.4	—	—

No significant group differences for MDA, SOD, CAT, GSH-Px, GSH, GST (all p > 0.05); GR, p < 0.001.

GR activity showed a significant group effect (p < 0.001). GR decreased from 153.4 nmol/min/g in the sham group to 21.7 nmol/min/g following I/R, corresponding to an 85.8% reduction. Memantine (21.8 nmol/min/g) and curcumin (22.9 nmol/min/g) did not restore GR activity. Caffeic acid demonstrated a partial increase (30.2 nmol/min/g); however, values remained substantially lower than those of the sham group.

Spearman correlation analysis demonstrated a strong negative association between GR and GST activities and histopathological damage scores (r = −0.71 to −0.82, p < 0.001). Conversely, positive correlations were identified between caspase-3, IL-1β, TNF-α immunoreactivity levels and MDA concentrations and the severity of neuronal injury (r = 0.68 to 0.79, p < 0.001).

Complete biochemical results (median and IQR values) are presented in Table 4.

## 4 Discussion

In this study, the effects of curcumin, memantine, and caffeic acid on cerebral ischemia reperfusion injury were examined through the combined evaluation of biochemical, histopathological, and immunohistochemical parameters. This combined evaluation allows structural tissue findings and molecular parameters to be interpreted together within the same study design. The findings indicate that the investigated agents exert differential effects on oxidative stress, inflammatory activity, and cell death related mechanisms.

Compared with the sham group, the I/R group demonstrated significant histopathological findings characterized by neuronal degeneration, neuronophagia, reactive gliosis, and vascular hyperemia (p < 0.001). In parallel, antioxidant enzyme activities were reduced and MDA concentrations were elevated. These results confirm that the model induced marked cortical oxidative stress and tissue injury. The morphological characteristics and distribution of cortical lesions are consistent with previous reports describing cortical susceptibility to ischemic injury (Subedi and Gaire, 2021; Cabalar et al., 2022).

In the memantine and caffeic acid treated groups, histopathological findings were significantly reduced compared with the I/R group, with scores approaching those of the sham group (p < 0.001). Decreases in IL-1β, TNF-α, Caspase-3, and TUNEL positivity were also observed (p < 0.001). In both groups, antioxidant enzyme activities showed higher values and MDA concentrations showed lower values relative to the I/R group. These findings indicate that memantine and caffeic acid are associated with reductions in oxidative stress, inflammatory activity, and apoptosis related processes in cortical tissue. Previous studies have reported that memantine limits excitotoxic neuronal injury (Chen and Lipton, 1997; Dogan et al., 1999), whereas caffeic acid reduces inflammatory cytokine expression and apoptosis related signaling (Zhao et al., 2017; Liang et al., 2015). The present findings are consistent with these reports.

In contrast, although curcumin treatment was associated with higher antioxidant enzyme activities compared with the I/R group, histopathological findings did not differ significantly from the I/R group (p > 0.05). Immunohistochemical analysis showed decreases in inflammatory mediators and apoptosis related indicators; however, these changes did not reach the same level observed in the memantine and caffeic acid groups. This indicates that improvement in biochemical antioxidant parameters may not necessarily correspond to structural tissue findings. Similar findings were reported by Bavarsad et al. (2019), who described dissociation between oxidative stress marker reduction and histopathological injury severity. Subedi and Gaire (2021) also reported that increases in antioxidant enzyme levels may not always be associated with reductions in neuronal degeneration and reactive gliosis. Zhang et al. (2023) further indicated that attenuation of inflammatory responses does not invariably result in measurable reduction of cortical tissue injury. The present findings are in agreement with these reports.

A strong inverse correlation was identified between GR and GST activities and histopathological damage scores (p < 0.001), indicating that decreases in these enzymes parallel increased tissue injury severity. In contrast, no significant correlation was observed between MDA levels and histological injury. This finding suggests that lipid peroxidation markers alone may be insufficient to accurately reflect cortical tissue injury.

Taken together, these findings highlight the importance of evaluating multiple interconnected pathways in the pathogenesis of cerebral ischemia rather than relying on a single biochemical indicator. Integrated assessment of oxidative stress, inflammatory mediators, and apoptosis-related processes provides a more comprehensive interpretation of tissue injury.

Treatment was initiated 30 min after reperfusion and continued for 5 days. Several previous investigations have implemented pre ischemic or prophylactic administration paradigms. For example, Bavarsad et al. (2019) primarily emphasized oxidative stress parameters, whereas Subedi and Gaire (2021) focused on antioxidant enzyme activity and inflammatory signaling. Zhang et al. (2023) examined inflammatory pathways but did not combine semi quantitative histopathological grading with biochemical evaluation in a single structured analysis. In contrast, the present study applied a defined post ischemic intervention protocol and assessed histopathological findings, immunohistochemical indicators, and biochemical enzyme activities within the same analytical framework. This approach provides a consistent basis for comparison of structural and molecular outcomes.

The three agents were administered individually to allow direct comparison within the same bilateral carotid occlusion model and grading system. In previous reports, memantine (Chen and Lipton, 1997; Dogan et al., 1999), caffeic acid (Zhao et al., 2017; Liang et al., 2015), and curcumin (Bavarsad et al., 2019; Subedi and Gaire, 2021) were investigated in independent experimental settings using different protocols and outcome measures, which makes direct comparison between studies more difficult. By examining all three compounds within the same study design, the present work allows evaluation of their tissue level and molecular effects within a single injury model.

Several earlier investigations, including those by Zhao et al. (2017) and Adefegha et al. (2023), have focused primarily on biochemical endpoints without combining them systematically with semi quantitative histopathological grading and apoptosis related indicators in the same analysis. In the current study, histopathological scoring was analyzed together with inflammatory mediators, apoptosis related indicators, and biochemical enzyme activities within a standardized sampling level.

However, factors that are essential for translation into clinical practice were not examined in this study. Pharmacokinetic characterization, dose response analysis, delayed treatment protocols, and long term outcome assessment were outside the scope of this work. In addition, although memantine is already approved for certain neurological conditions, caffeic acid and curcumin do not yet have standardized formulations or established dosing strategies for ischemic applications. Further experimental and pharmacological investigations are therefore required before consideration of clinical use.

Overall, memantine and caffeic acid were associated with significant reductions in histopathological findings at the cortical level, whereas curcumin did not demonstrate a comparable degree of structural effect under the applied dose and treatment duration. These results suggest that evaluation of neuroprotective potential should extend beyond antioxidant enzyme activity and also include inflammatory mediators and apoptosis related indicators.

## Data Availability

The raw data supporting the conclusions of this article will be made available by the authors, without undue reservation.

## Supplementary material

SUPPLEMENTARY FIGURE S1 shows the schematic coronal map (bregma –1.8 to –3.0 mm) illustrating the cortical sampling region analyzed in the study. Supplementary Material should be uploaded separately on submission, if there are Supplementary Figures, please include the caption in the same file as the figure. Supplementary Material templates can be found in the Frontiers Word Templates file.
