# Supplementary figures and images for: Neuroprotective effects of curcumin, memantine, and caffeic acid in a Rat model of cerebral ischemia-reperfusion injury
Source: Front Pharmacol. 2026 Mar 13;17:1739566. doi: 10.3389/fphar.2026.1739566 (PMC13021649; doi:10.3389/fphar.2026.1739566)

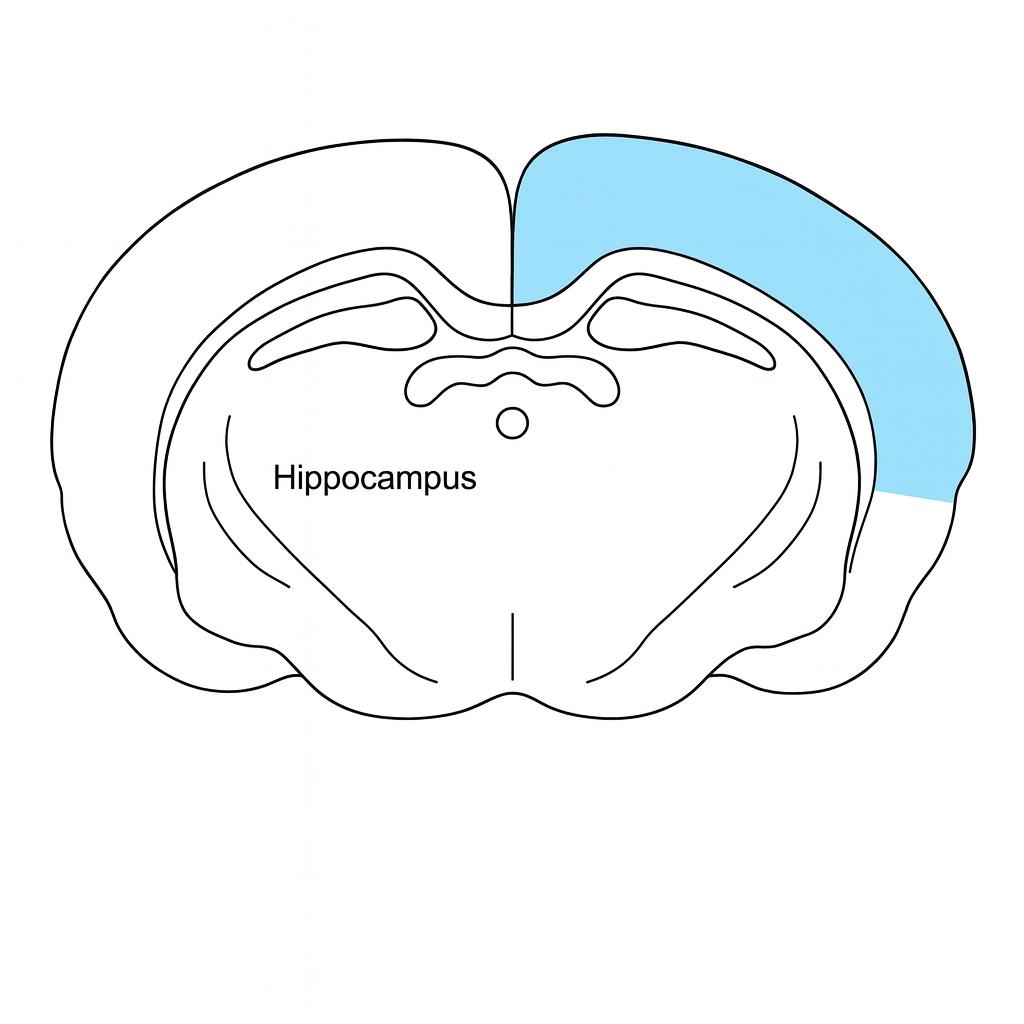

Supplement: Supplementary file 1 [file Image1.jpeg]
